# Dynamic Effects of Aortic Arch Stiffening on Pulsatile Energy Transmission to Cerebral Vasculature as A Determinant of Brain-Heart Coupling

**DOI:** 10.1038/s41598-020-65616-7

**Published:** 2020-05-29

**Authors:** Arian Aghilinejad, Faisal Amlani, Kevin S. King, Niema M. Pahlevan

**Affiliations:** 1 Department of Aerospace and Mechanical Engineering, University of Southern California, Los Angeles, CA USA; 2 Huntington Medical Research Institutes, Advanced Imaging Center, Pasadena, CA USA; 3 Division of Cardiovascular Medicine, Keck School of Medicine, University of Southern California, Los Angeles, CA USA

**Keywords:** Biomedical engineering, Mechanical engineering, Neurodegenerative diseases, Cardiovascular biology

## Abstract

Aortic stiffness increases with age and is a robust predictor of brain pathology including Alzheimer’s and other dementias. Aging causes disproportionate stiffening of the aorta compared with the carotid arteries, reducing protective impedance mismatches at their interface and affecting transmission of destructive pulsatile energy to the cerebral circulation. Recent clinical studies have measured regional stiffness within the aortic arch using pulse wave velocity (PWV) and have found a stronger association with cerebrovascular events than global stiffness measures. However, effects of aortic arch PWV on the transmission of harmful excessive pulsatile energy to the brain is not well-understood. In this study, we use an energy-based analysis of hemodynamic waves to quantify the effect of aortic arch stiffening on transmitted pulsatility to cerebral vasculature, employing a computational approach using a one-dimensional model of the human vascular network. Results show there exists an optimum wave condition—occurring near normal human heart rates—that minimizes pulsatile energy transmission to the brain. This indicates the important role of aortic arch biomechanics on heart-brain coupling. Our results also suggest that energy-based indices of pulsatility combining pressure and flow data are more sensitive to increased stiffness than using flow or pressure pulsatility indices in isolation.

## Introduction

Neurodegenerative diseases such as Alzheimer’s and other related dementias have reached an epidemic proportion with a significant impact on public health. In 2017, it is estimated that there were 5.7 million Americans with Alzheimer’s disease (AD) which was associated with a cost of 232 billion dollars for care and lost productivity for patients and their caregivers^1^. There is no cure for AD and there are different major risk factors recognized for Alzheimer development such as increasing age, family history, hypertension, hypotension, and high cholesterol levels^2^. Although the cause or cure for AD is not fully understood, the identification of the risk factors that cause brain injury may offer important ways to mitigate the development of this disease^3^. Vascular risk factors such as hypertension increase the risk for AD and serve as potential targets for prevention^4^. Recent studies have demonstrated that identifying arterial stiffening can improve the prediction of hypertension-related risk for both the cerebral microvascular ischemic disease and neurodegenerative changes associated with AD^5^. In a recent review study, Stone *et al*.^6^ have demonstrated that age-related dementia results from the destructive impact of the pulse on cerebral vasculature which, in turn, can be attributed to the age-related stiffening of the aorta.

Aortic stiffness increases with age and is one of the earliest pathological changes within the arterial wall. The proximal aorta acts as a coupling device between the heart and the brain, regulating the amount of pressure and flow pulsatility transmitted to the cerebral vasculature^7^. In young healthy adults, the conduit arteries arising from the aorta, such as the carotid artery, have higher stiffnesses than the aortic arch. This distinct difference in compliance between the highly elastic aorta and the more muscular branch vessels results in a high impedance mismatch. This mismatch causes a large pulse wave reflection at the aorta-carotid interface which protects the brain microvasculature from high pulsatile energy. Disproportionate age-related stiffening of the aorta (relative to the carotid arteries) is theorized to reduce this protective impedance mismatch at the interface and thereby affect the wave reflection^8^. These age-related changes in the biomechanics of the aorta may therefore have a significant impact on the transmission of potentially deleterious pulsatile energy into the microcirculation resulting in impaired regulation of local blood flow and in tissue damage. The brain is particularly susceptible to pulsatile damage resulting from low vascular resistance related to its high resting rate of blood flow and low impedance^9^. Through a study of the pressure and flow waveform in over 1000 normal subjects, Kim *et al*.^10^ have shown that the cerebral microvascular damage in older patients can be attributed to the tearing of delicate media by high pulsatile pressure (causing hemorrhage) and to the dislodging of endothelial cells (causing thrombosis and microinfarcts).

In cerebral circulation, a common manifestation of hypertensive damage is white matter hyperintensities (WMH)^11^. WMH predict risk for significant morbidity with aging including risk of death, functional impairment and dementia^12,13^. In the population-based Dallas heart study^14,15^ led by one of the authors of this manuscript, K.S.K, it was shown that aortic arch pulse wave velocity (PWV) measured by magnetic resonance is a more a robust predictor of WMH volume than clinical assessments of blood pressure or hypertension. Furthermore, the presence of aortic stiffness was independent of—but additive to—the presence of hypertension in predicting WMH^14^. These results indicate the existence of a potential link between arterial stiffening and AD. While there are several biomarkers for quantification of arterial stiffness such as total arterial compliance and carotid-femoral PWV, recent clinical data reveals a stronger association of aortic arch PWV with cerebrovascular and other extra-cardiac events^15^. However, to the best of our knowledge, the effect of aortic arch stiffening on transmitted pulsatility to the brain and its linking mechanism has not been investigated in a systems-based approach. Such a method enables evaluation of the potential interactions and contributions to wave dynamics from other important hemodynamic parameters such as the heart rate (HR).

There is an essential need for a well-designed quantitative study to investigate the association between aortic arch stiffness and the pulsatile energy transmission to the cerebrovascular network (that causes brain insult) in order to identify potential therapeutic targets for intervention. Hence, in this work, we are interested in studying how changes in aortic stiffness and their corresponding wave dynamics lead to excessive pulsatile energy transmission to the brain. To this end, we have employed a one-dimensional (1D) computational model of the arterial network that has been validated and used extensively for studying the pressure and flow wave propagation along the arterial network^16–19^.

In clinical studies, flow and pressure pulsatility indices in the carotid arteries are commonly-used parameters for determining pulsatility transmission to the brain^20^. In this study, we have performed an energy-based analysis for quantification of the hemodynamic pulsatility. Specifically, we have focused on the pulsatile portion of the net power transmitted to the brain. In the Dallas Heart Study^14^, it has been shown that aortic arch PWV (which is a contributor to the pulsatile portion of the net power) and increased blood pressure (which is a contributor to the steady portion of the power) have independent associations with brain vascular insult. Due to this independence, we put the focus of the current study only on the pulsatile portion of the transmitted power in order to capture the dynamic effects of arterial stiffening on pulsatility transmitted to the cerebral vasculature. This analysis considers the combined effects of flow and pressure propagation into the brain and demonstrates greater sensitivity to increased stiffness than using a pressure or flow pulsatility index alone. Simulations have been performed at different HRs to account for different wave conditions^21,22^.

### Theoretical indicators of an optimum wave condition

As an introduction to the energy-based analysis used in this paper, it is helpful to consider the theoretical relation for the transmitted power through waves. Following the analysis of Alastruey *et al*.^23^ and Passerini^24^, the governing equations for pressure and flow propagation that are employed later in this work can be linearized about a reference state in the space-time domain. Under the assumption of periodicity for the propagated pressure and flow waves inside the vasculature, the solutions for pressure *p*(*x*, *t*) and flow *q*(*x*, *t*) at a time *t* and at a position *x* can be sought as harmonic waves of the form

1a $$p(x,t)={p}_{0}{e}^{i(\omega t-kx)},$$

1b $$q(x,t)={q}_{0}{e}^{i(\omega t-kx)},$$

where ${p}_{0}$ and ${q}_{0}$ are the (possibly complex) amplitudes of the pressure and flow at $(x,t)=(0,0)$, $\omega$ is the frequency of the oscillation, and *k* is the wavenumber. It can be shown^23^ that for a wavenumber *k*, the corresponding dispersion relation for temporal frequency $\omega$ is given by

2 $$\omega =\frac{i({C}_{2}{k}^{2}+{C}_{3})\pm \sqrt{-({C}_{2}{k}^{2}+{C}_{3}{)}^{2}+4{C}_{1}{k}^{2}}}{2}$$

and the subsequent phase velocity *c*_*w*_ = *ω*/*k* (of particular interest in this work) is given by

3 $${c}_{w}=\frac{i({C}_{2}k+\frac{{C}_{3}}{k})\pm \sqrt{-{\left({C}_{2},k,+,\frac{{C}_{3}}{k}\right)}^{2}+4{C}_{1}}}{2},$$

where $i=\sqrt{-1}$ and where the constants *C*_1_, *C*_2_, and *C*_3_ are combinations of physical parameters that ultimately account for wall compliance, flow inertia and the resistance to the flow.

Assuming that pressure amplitude *p*_0_ is real-valued, a mass conversation argument yields a resulting complex amplitude for flow as a function of phase velocity^23^, i.e., ${q}_{0}={q}_{0}({c}_{w})$. Hence the corresponding physical solutions, which are represented by the real parts of Eq. (1a) and Eq. (1b), are given by

4a $$\Re (p(x,t))={p}_{0}{e}^{\Im (k)x}\,\cos (\omega t-\Re (k)x),$$

4b $$\Re (q(x,t))={e}^{\Im (k)x}(\Re ({q}_{0}({c}_{w}))\cos (\omega t-\Re (k)x)-\Im ({q}_{0}({c}_{w}))\sin (\omega t-\Re (k)x)),$$

where $\Re$ and $\Im$ denote the real and imaginary parts of a complex number, respectively. The complex-valued amplitude of the flow given by Eq. (1b) indicates a (frequency-dependent) phase-shift between the pressure *p* and the flow *q* that is expressed in Eq. (4b). The corresponding instantaneous transmitted power, derived by multiplying the propagated pressure and flow at a point *x*, is hence given by

5 $$\dot{W}=\Re (p(x,t))\cdot \Re (q(x,t))=\frac{1}{2}{p}_{0}\Re ({q}_{0}({c}_{w})){e}^{2\Im (k)x}+\frac{1}{2}{p}_{0}|{q}_{0}({c}_{w})|{e}^{2\Im (k)x}\,\sin (2\omega t-2\Re (k)x+\varphi ({c}_{w})),$$

where $|{q}_{0}({c}_{w})|$ denotes the complex amplitude of the flow rate and $\varphi ({c}_{w})$ is a (frequency-dependent) phase difference.

Even for the linearized system of governing equations considered in the above analysis, one can note the complex (nonlinear) dependencies on the wavenumber for oscillation frequency $\omega$ (Eq. (2)) and for wave speed ${c}_{w}$ (Eq. (3)). In particular, these dependencies are prominent in the corresponding transmitted power given by Eq. (5); they may be reasonably assumed to be further nonlinear when incorporating more elastic/viscoelastic effects as well as wave reflections that result from interfaces in vasculature (see Methods). This very simplified analysis suggests that extrema may be found in pulsatility power curves as a function of both frequency (i.e., HR) and wave speed (i.e., PWV). This seems to be the case even in the absence of wave reflections (i.e., branching of the vasculature) and the fully-coupled nonlinear formulation considered later in this paper. Thus, the primary motivation of this work is to investigate these dynamic effects through a computational approach that treats a more physiologically relevant and physically accurate viscoelastic hemodynamics model for understanding the behavior of these physical variables on energy transmission to the brain.

All in all, wave dynamics in a compliant tube depend on three parameters: 1) fundamental frequency of the propagating waves, 2) wave speed as a function of material properties, and 3) reflection sites^25^. In what follows, we have focused primarily on investigating the effect of the first two parameters on the transmitted pulsatile power to the brain. However, the relative change of aortic arch stiffness with respect to the branches will affect the wave reflections as well, and hence the third is implicitly studied through the physiological relevance of the physical and mathematical models.

## Methods

### Physical model

**Figure 1:**
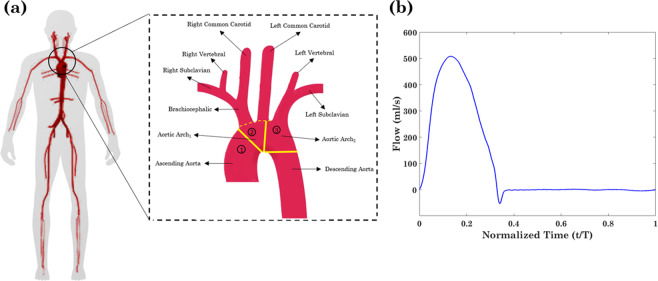
(**a**) Schematic of the systemic vasculature with a zoom on the aortic arch (dashed box). (**b**) The physiological inflow waveform prescribed at the aortic root of the baseline model where the corresponding cardiac output is 5.2 l/min.

A validated 1D model of the vascular network based on space-time variables has been employed in this study^26,27^. 1D arterial models have been shown to be a powerful tool for studying hemodynamics and wave dynamics in both large systemic arteries^28^ as well as the entire adult circulation^29^. The physical model used in this study consists of 55 larger systemic arteries, where each artery is modeled as a visco-elastic tube characterized by its diameter, length, Young’s modulus, viscosity and wall thickness. To consider the effect of viscoelasticity^30^, the Voigt-type model (a combination of a linear spring and a linear viscous dashpot connected in parallel^31^) has been employed. The arterial wall is assumed to be thin, incompressible, homogenous and isotropic. In this study, our focus was to investigate the effect of proximal aorta stiffness on pulsatility transmission into the brain. For this purpose, the first three portions of the aorta (1-ascending aorta, 2-proximal aortic arch feeding the brachiocephalic and left common carotid arteries, and 3-distal aortic arch feeding the left vertebral and left subclavian arteries) were altered while all the other segments of the aorta and systemic vasculature were kept constant (Fig. 1(a)).

At the inlet, we impose a physiological flow wave at the aortic root as shown in Fig. 1(b). The blood is assumed to be an incompressible Newtonian fluid with density of *ρ* = 1050 kg/m^3^ and viscosity of *µ* = 4 mPa∙s. Different levels of aortic arch rigidity are considered by employing multiplicative factors of a minimum rigidity level ${E}_{1}(x)$ that corresponds to the baseline PWV of *c*_1_ initially prescribed to the model. The baseline properties for aortic segments that were altered in this study are presented in Table 1. In this table, ${{\boldsymbol{c}}}_{{\boldsymbol{in}}}$ and ${{\boldsymbol{c}}}_{{\boldsymbol{out}}}$ refer to the wave speed at the inlet and outlet of the segment, respectively.

**Table 1:** The baseline values of the physical characteristics for the relevant arterial segments.

Name	Length (cm)	${{\boldsymbol{c}}}_{{\boldsymbol{in}}}{\boldsymbol{\to }}{{\boldsymbol{c}}}_{{\boldsymbol{out}}}\,\left(\frac{{\boldsymbol{m}}}{{\boldsymbol{s}}}\right)$
Ascending Aorta	5.8	$3.95\to 3.96$
Aortic Arch_1_	2.3	$4.15\to 4.2$
Aortic Arch_2_	4.5	$4.35\to 4.39$
Right Common Carotid	10.8	$5.32\to 6.47$
Right Vertebral	17.1	$8.03\to 8.73$
Left Common Carotid	16	$5.51\to 6.78$
Left Vertebral	17	$8.03\to 8.73$

Values are adopted from Alastruey^35^.

### Mathematical and computational model

Conservation of mass and momentum applied to a 1D impermeable and deformable tubular control volume of an incompressible Newtonian fluid, flowing with a constant axisymmetric velocity profile, yields the system of equations

6a $$\{\frac{\partial A}{\partial t}+\frac{\partial (AU)}{\partial x}=0,\,$$

6b $$\{\frac{\partial U}{\partial t}+U\frac{\partial U}{\partial x}+\frac{1}{\rho }\frac{\partial p}{\partial x}=\frac{f}{\rho A},$$

where *x* is the axial coordinate along the vessel, *t* is the time, *A(x, t)* is the cross-sectional area of the lumen, *U(x,t)* is the average axial velocity, *p(x, t)* is the average internal pressure over the cross-section, and *f* is the friction force per unit length. For mathematical simplification it is assumed that the Coriolis coefficient (velocity shape factor) is unity, resulting in a flat velocity profile for the 1D model and hence a corresponding friction force per unit length of $f=-\,22\mu \pi U$^32^. In order to close the system of Eq. (6) for the three unknowns *A(x, t)*, *U(x, t)* and *p(x, t)*, a constitutive relation between the sectional pressure *p* and area *A* can be implemented by a Voigt-type viscoelastic tube law. This relationship accounts for the FSI of the problem and can be derived as^33^

7 $$p={p}_{{\rm{ext}}}+\frac{G(x)}{{A}_{0}}(\sqrt{A}-\sqrt{{A}_{0}})+\frac{\lambda (x)}{{A}_{0}\sqrt{A}}\frac{\partial A}{\partial t},$$

where ${p}_{{\rm{ext}}}$ is the constant external pressure and ${A}_{0}$ is the constant cross-sectional area at equilibrium state (*p*, *U*) = (*p*_ext_, 0). The spatially-varying functions $G(x)$ and $\lambda (x)$ are related to the elastic and visco-elastic properties of the arterial wall, respectively, and can be given in terms of material properties as

8 $$G(x)=\frac{\sqrt{\pi }E(x)h(x)}{(1-{{\vartheta }}^{2})},$$

9 $$\lambda (x)=\frac{\sqrt{\pi }\varphi (x)h(x)}{2(1-{{\vartheta }}^{2})},$$

where *E(x)* is the Young’s modulus, $\varphi (x)$ is the vessel wall viscosity, *h(x)* is the wall thickness, and ${\vartheta }$ is the Poisson’s ratio of the wall (taken to be ${\vartheta }=1/2$ assuming the wall is incompressible). Note that following the definition of the local PWV in terms of area and pressure, the wave speed can be written in terms of the elasticity factor $G(x)$ as

10 $$c=\sqrt{\frac{A}{\rho }\frac{\partial p}{\partial A}}=\sqrt{\frac{G(x)}{2\rho {A}_{0}}}{A}^{1/4}$$

The system of partial differential equations (PDEs) in Eqs. (6 and 7) can be represented in matrix form as

11a $$\{\frac{\partial {\rm{U}}}{\partial t}+\frac{\partial {\rm{F}}}{\partial x}={{\rm{H}}}_{U},$$

11b $$\{U=[\begin{array}{c}A\\ U\end{array}],\,{{\rm{H}}}_{U}=\left[\begin{array}{c}0\\ \frac{f}{\rho A}\end{array}\right],$$

11c $$\{F={{\rm{F}}}_{e}+{{\rm{F}}}_{v},\,\left[\begin{array}{c}AU\\ \frac{{U}^{2}}{2}+\frac{{p}_{ext}+\frac{G(x)}{{A}_{0}}(\sqrt{A}-\sqrt{{A}_{d}})}{\rho }\end{array}\right]+\left[\begin{array}{c}0\\ -\frac{\lambda (x)}{{A}_{0}\sqrt{A}}\frac{\partial (AU)}{\partial x}\end{array}\right],$$

where Eq. (6a) has been used to replace $\partial A/\partial t$ with $\partial (AU)/\partial x$ in Eq. (11c). In order to solve this system of hyperbolic PDEs, a discontinuous Galerkin scheme can be employed for simplicity and fast convergence without causing excessive dispersion or diffusion errors^34^. Consider a spatial domain $\Omega$ = (*a*, *b*) discretized into a mesh of ${N}_{el}$ elemental non-overlapping regions ${\Omega }_{e}=({x}_{e}^{l},{x}_{e}^{u})$, *e* = 1, …, $\,{N}_{el}$, where ${x}_{e}^{u}={x}_{e+1}^{l}$ and $\cup \frac{{N}_{el}}{e=1}{\Omega }_{e}=\Omega$. The weak form of Eq. (11a) is given by

12 $${\left(\frac{\partial {\rm{U}}}{\partial t},\varphi \right)}_{\Omega }+{\left(\frac{\partial {\rm{F}}}{\partial x},\varphi \right)}_{\Omega }={({{\rm{H}}}_{U},\varphi )}_{\Omega },$$

where $\varphi (x)$ is an arbitrary function on the domain $\Omega$ and ${(v,u)}_{\Omega }={\int }_{\Omega }uvdx$ is the standard ${L}^{2}(\Omega )$ inner product. The discrete form of the conservative representation in Eq. (12) can be given by^35^

13 $$\mathop{\sum }\limits_{e=1}^{{N}_{el}}\left[{\left(\frac{\partial {{\rm{U}}}^{\delta }}{\partial t},{\varphi }^{\delta }\right)}_{{\Omega }_{e}}+{\left(\frac{\partial {\rm{F}}({{\rm{U}}}^{\delta })}{\partial x},{\varphi }^{\delta }\right)}_{{\Omega }_{e}}+{[{\varphi }^{\delta }\cdot \{{{\rm{F}}}^{u}-{\rm{F}}({{\rm{U}}}^{\delta })\}]}_{{x}_{e}^{l}}^{{x}_{e}^{u}}\right]=\mathop{\sum }\limits_{e=1}^{{N}_{el}}{({{\rm{H}}}_{U}^{\delta },{\varphi }^{\delta })}_{{\Omega }_{e}},$$

where, following a traditional Galerkin approach, the superscript $\delta$ indicates that the variable is approximated in the finite space of piecewise polynomial vector functions (the trial space) and ${{\rm{F}}}^{u}$ is the approximation of the flux at an interface. In the trial space, the expansion basis is chosen to be Legendre polynomials due to their orthogonality with respect to the ${L}^{2}({\Omega }_{e})$ inner product. Hence, the approximated solution on each elemental region ${{\rm{U}}}_{e}^{\delta }$ can be expanded to order *M* as

14 $${{\rm{U}}}_{e}^{\delta }({\chi }_{e}(\xi ),t)=\mathop{\sum }\limits_{j=0}^{M}{L}_{j}(\xi ){\hat{{\rm{U}}}}_{e}^{j}(t),$$

where ${L}_{j}(\xi )$ is the Legendre polynomial of order *j* with corresponding time-varying coefficient ${\hat{{\rm{U}}}}_{e}^{j}(t)$, and ${\chi }_{e}(\xi )={x}_{e}^{L}(1-\xi )/2+{x}_{e}^{R}(1+\xi )/2$ is the elemental mapping. Substituting Eq. (14) into Eq. (13) and letting ${{\rm{\varphi }}}_{e}^{\delta }={{\rm{U}}}_{e}^{\delta }$ yields a system of *M*+*1* ordinary differential equations (ODEs) for each $\,{\hat{{\rm{U}}}}_{e}^{j}(t)$, *j* = *0*, …, *M* as

15 $$\frac{d{\hat{{\rm{U}}}}_{e}^{j}(t)}{dt}=\psi ({{\rm{U}}}_{e}^{\delta })=-{\left(\frac{\partial {\rm{F}}({{\rm{U}}}^{\delta })}{\partial x},{L}_{j}\right)}_{{\Omega }_{e}}-\frac{2}{{x}_{e}^{R}-{x}_{e}^{L}}{[{L}_{j}\cdot \{{{\rm{F}}}^{u}-{\rm{F}}({{\rm{U}}}^{\delta })\}]}_{{x}_{e}^{L}}^{{x}_{e}^{R}}+{({{\rm{H}}}_{U}^{\delta },{L}_{j})}_{{\Omega }_{e}}.$$

Solving the ODE in Eq. (15) for each ${\hat{{\rm{U}}}}_{e}^{j}(t)$ yields the coefficients required to reconstruct the physical solution given by Eq. (14). To calculate the fluxes at each interface between elements, ${{\rm{F}}}^{u}$ is decomposed into the elastic term ${{\rm{F}}}_{e}^{u}$ and the viscous term ${{\rm{F}}}_{v}^{u}$. The elastic term is determined by solving the Riemann problem and the viscous term can be treated by the average of the lower and upper limits in an elemental region. In order to numerically resolve ${\hat{{\rm{U}}}}_{e}^{j}(t)$ at a discrete time $t$ = ${t}_{n+1}$, a second-order Adams-Bashforth time integration scheme is applied to Eq. (15) for each *j* = 0, …, *M* and *e* = 1, …, $\,{N}_{el}\,$. This yields the iterative sequence with a time-step $\Delta t\,$^35^

16 $${({\hat{{\rm{U}}}}_{e}^{j}({t}_{n+1}))}^{n+1}=({\hat{{\rm{U}}}}_{e}^{j}({t}_{n}))+\frac{3\Delta t}{2}\psi ({{\rm{U}}}_{e}^{\delta }({t}_{n}))-\frac{\Delta t}{2}\psi ({{\rm{U}}}_{e}^{\delta }({t}_{n-1})),$$

where we have taken the notational license ${{\rm{U}}}_{e}^{\delta }(t)={{\rm{U}}}_{e}^{\delta }({\chi }_{e}(\xi ),t)$.

A physiological flow wave (Fig. 1(b)) has been applied as an inlet flow to the aortic root and scaled to give a cardiac output of 5.2 l/min for any given HR. At arterial segment junctions and bifurcations (Fig. 1(a)), the boundary conditions are prescribed by enforcing conservation of mass and continuity of the total pressure $p+.5\rho {U}^{2}$. By decomposing the governing system of equations (Eq. (6a,b)) into the characteristic variables, the system can be interpreted in terms of forward and backward traveling waves. At any bifurcations and junctions, we have six unknowns: (${A}_{p},{U}_{p}$) in the parent vessel, (${A}_{d1},{U}_{d1}$) in its first daughter vessel and (${A}_{d2},{U}_{d2}$) in its second daughter vessel. This a set of six independent equations. Within the parent vessel, the information can only reach the bifurcation by the forward traveling wave, while within the daughter vessels the information can only reach the bifurcation by the backward traveling wave. Therefore, the first three equations can be obtained by imposing that the characteristic variables in each vessel (parent and daughters) remain constant ($dW/dt=0$, where *W* is the characteristic variable)^35^. The other three independent equations can be obtained by requiring the conservation of mass and continuity of the momentum balance. The latter condition leads to continuity of the total pressure at the boundary. It has been shown that energy losses at arterial segment junctions only change the mean pressure and flow by less than 0.5%^36^, hence they are disregarded in the model. Finally, at terminal boundaries, three-element RCR Windkessel models are employed as 0D lumped parameters that act as the outflow boundary condition on pressure *p*(*t*) and flow *q*(*t*) = *AU* at each peripheral branch. This is given by the ODE

17 $$\frac{dp}{dt}={R}_{1}\frac{dq}{dt}+\frac{1}{{R}_{2}C}(({R}_{1}+{R}_{2})q-p),$$

for inflow resistance R_1_ (that matches the characteristic impedance of the terminal vessel), peripheral compliance C and outflow resistance R_2_—all of which are chosen within the average physiological range^37^.

A validated code called “Nektar” was used to solve the discretized equation ^16,33,38–41^. This code has been developed for solving the nonlinear 1D equations of blood flow in a given network of compliant vessels subjected to boundary and initial conditions. Importantly, the code has been validated against *in vitro*^33,36^ and *in vivo*^28,39,42^ experiments. A Linux operating system has been used to compile the code on a standalone workstation equipped with an Intel Core i7 CPU (6 cores and 3201 MHz) with 32GB memory. Each simulation is run at a time-step of $\Delta t=10\,\mu {\rm{s}}$. At least 10 cardiac cycles are simulated in order to ensure that a periodic steady state is reached. The results are then processed and analyzed using MATLAB (The MathWorks, Inc., MA, USA).

### Hemodynamic analysis

The total power ${\bar{P}}_{total}$ transmitted to the brain over a cardiac cycle of length *T* is calculated as the average of the product of the pressure *p(t)* and the flow *q(t)* in each brain segment. The steady power ${\bar{P}}_{s}$ is computed as the product of mean pressure ${p}_{mean}$ and mean flow ${q}_{mean}$ in each segment. The pulsatile transmitted power ${\bar{P}}_{pulse}$ is the difference between the total power and the steady power. Each of these power quantities are respectively given by

18 $${\bar{P}}_{total}=\frac{1}{T}{\int }_{0}^{T}p(t)q(t)dt,$$

19 $${\bar{P}}_{s}={p}_{mean}{q}_{mean},$$

20 $${\bar{P}}_{pulse}={\bar{P}}_{total}-{\bar{P}}_{s}.$$

Based on the above equations, the pulsatile power percentage *PPP* is defined as the ratio between the pulsatile transmitted power and the total power, i.e.,

21 $$PPP=\frac{{\bar{P}}_{pulse}}{{\bar{P}}_{total}}.$$

Common clinical parameters such as flow pulsatility index *FPI* and pressure pulsatility index *PPI*^20^ are defined as

22 $$FPI=\frac{{q}_{max}-{q}_{min}}{\frac{1}{T}{\int }_{0}^{T}q(t)dt},$$

23 $$PPI=\frac{{p}_{max}-{p}_{min}}{\frac{1}{T}{\int }_{0}^{T}p(t)dt},$$

where ${q}_{min}\,{\rm{and}}\,{q}_{max}$ (resp. ${p}_{min}\,{\rm{and}}\,{p}_{max}$) are the minimum and maximum flow (resp. pressure) transmitted to the brain during a cardiac cycle.

## Results

Simulations are run for five different levels of aortic arch PWV, starting from the baseline PWV of a healthy individual (c_1_) and moving towards different levels by multiplicative factors of c_1_ given by c_2_ = 1.25c_1_, c_3_ = 1.5c_1_, c_4_ = 2c_1_, c_5_ = 3c_1_ (see values in Table 1). Each case has been run for eight HRs (30, 47, 63, 75, 94, 126, 150, and 189 beats per minute (bpm)). In all simulations, the Cardiac Output (CO), the peripheral resistance (PR), the terminal compliance, the shape of the inflow wave and all the outflow boundary conditions are kept constant.

### Physiological accuracy of the model

**Figure 2:**
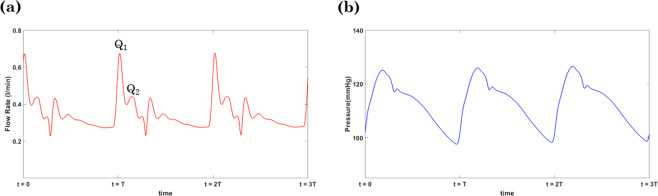
The simulated flow (**a**) and pressure (**b**) in the left common carotid artery at the baseline aortic arch PWV (see Table 1) and HR of 75 bpm.

A sample of flow and pressure in the left common carotid artery is shown in Fig. 2. The expected fiducial features of pressure and flow waveforms, including the pressure inflection point, the pressure dicrotic notch, and the peaks of the flow (Q_1_ and Q_2_), can be seen in Fig. 2(a,b).

**Figure 3:**
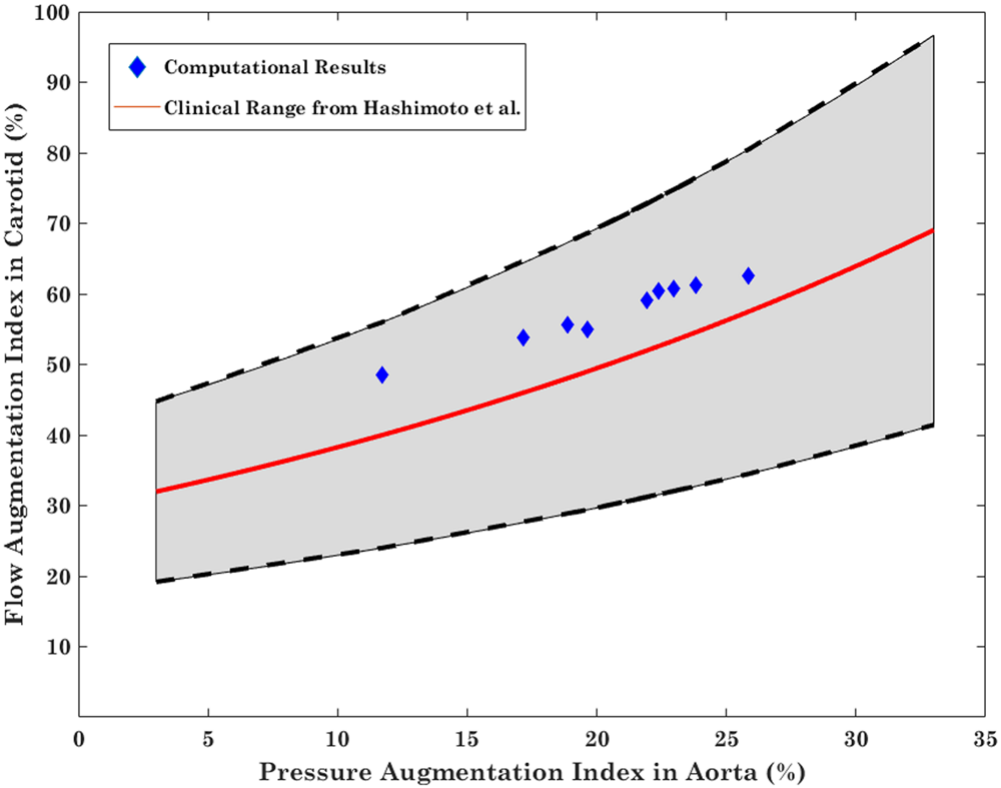
The carotid flow augmentation index versus the aortic pressure augmentation index. Red is the exponential fitted curve (r = 0.71) on the clinical data produced by Hashimoto *et al*.^20^. Blue diamonds represent simulation results for the baseline HR. The dashed upper and lower curves are based on the error bars reported by Hashimoto *et al*.^20^.

Figure 3 demonstrates the comparison between our simulated data with clinical data (reproduced from data by Hashimoto *et al*.^20^). The clinical data consists of recorded Doppler waveforms in 286 patients with hypertension in order to measure the carotid flow augmentation index defined as the ratio of late systolic flow height (Q_2_-Q_min_) over early systolic wave height (Q_1_-Q_min_). Pressure augmentation indices (augmented pressure over the maximum height of the pressure waveform) are computed from Tonometric pressure waveforms. The red curve in Fig. 3 is the exponential fitted curve (r = 0.71) on clinical data^20^. The dashed upper and lower curves are based on the error bars reported by Hashimoto *et al*.^20^.

### Effect of heart rate on transmitted pulsatility to the brain

**Figure 4:**
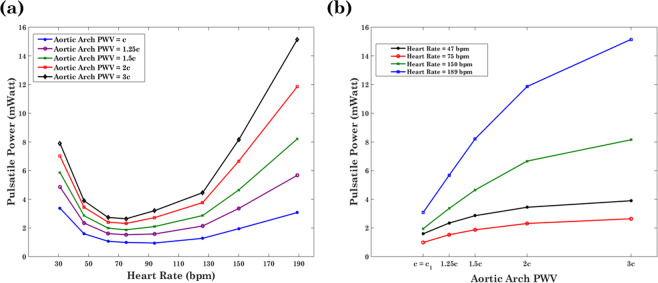
Average transmitted pulsatile power to the brain per cardiac cycle versus (**a**) the HR at different levels of aortic arch stiffness (PWV) and versus (**b**) the PWV at different HRs.

Figure 4(a) gives ${\bar{P}}_{pulse}$ computed by Eq. (20) as a function of HR for different levels of the aortic arch PWV. As mentioned previously, the CO is kept constant for all cases. Results are computed from the left common carotid artery hemodynamic waveforms (the only cerebral branch that is directly connected to the aortic arch). As HR increases, the value of ${\bar{P}}_{pulse}$ decreases until the HR reaches an optimum point where ${\bar{P}}_{pulse}$ is minimized. ${\bar{P}}_{pulse}$ increases with HR beyond this optimum point (Fig. 4(a)). Note that this phenomenon is present for all different multiplicative factors of aortic arch PWV. Figure 4(b) demonstrates the pulsatile power as a function of the aortic arch PWV at different HRs. As expected, pulsatile power in the carotid artery increases at all HRs when the aortic arch PWV increases.

**Figure 5:**
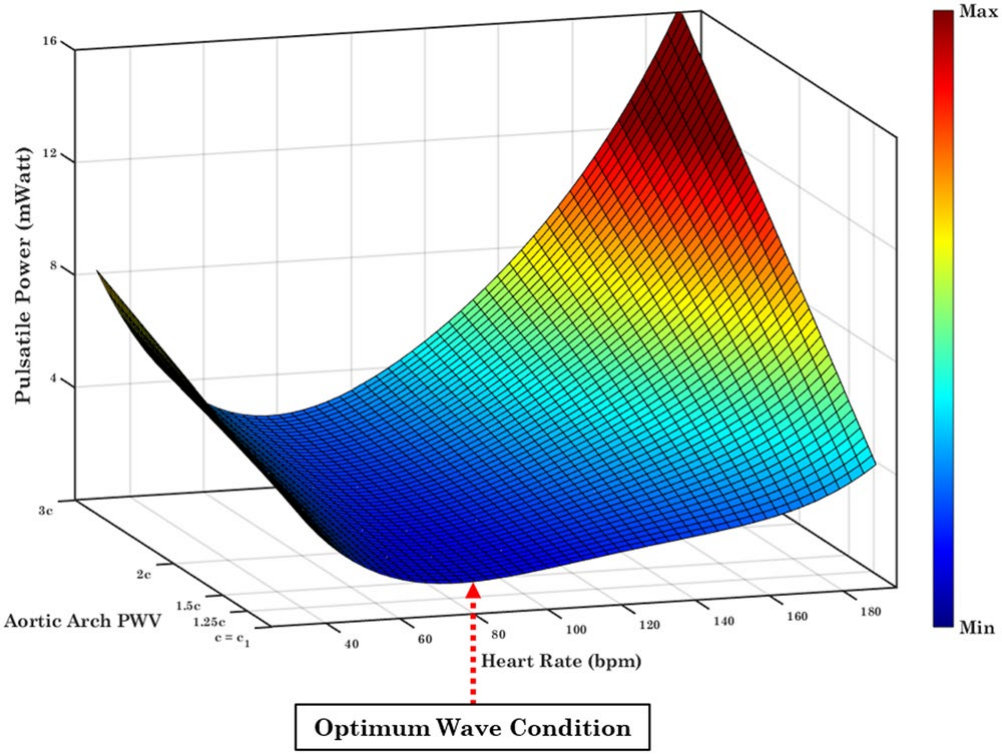
Pulsatile power transmission to the brain as a function of the Heart Rate and Aortic Arch PWV. Red arrow indicates the optimum region in which pulsatile power is minimized. The full 3D figure is provided as online supplementary material.

Figure 5 depicts a 3D interpolation mapping of the pulsatile power with respect to the aortic arch stiffness (as measured by PWV) and the HR. There is an optimum wave condition region in which pulsatile power transmission is minimized (red arrow in Fig. 5). This optimal region occurs at the baseline aortic arch PWV (for a normal adult) around a value of HR = 75 bpm.

### Flow and pressure pulsatility indices versus pulsatile power percentage

**Figure 6:**
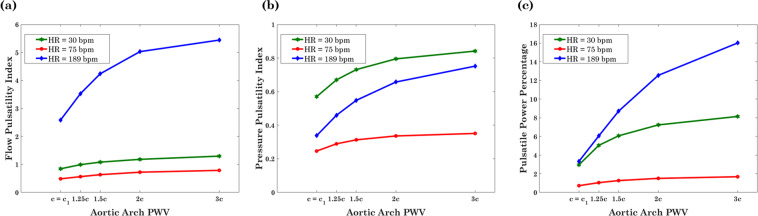
Flow Pulsatility Index (**a**), Pressure Pulsatility Index (**b**), and Pulsatile Power Percentage (**c**) within the left common carotid artery at different aortic arch PWVs for a normal HR (red), the lowest investigated HR (green) and the highest investigated HR (blue).

Figure 6 compares pulsatility indices and PPP at different aortic arch PWVs for three cases of the HR: (1) a normal heart rate, (2) the highest simulated heart rate (HR = 189 bpm), and (3) the lowest simulated heart rate (HR = 30 bpm). This range of HRs covers a large domain of different wave conditions.

## Discussion

In this study, we have employed a physiologically accurate computational model of the systemic vasculature to investigate the effect of aortic arch stiffening on the transmission of excessive wave pulsatility to the cerebral circulation. Our results suggest that: (1) there exists an optimum wave condition in the aorta that minimizes the harmful pulsatile energy (power) transmission to the brain, (2) at different wave conditions (i.e. different HR and aortic arch PWV), this optimum wave condition occurs around a value near the normal human HR (75 bpm), and (3) an index based on pulsatile power (i.e., a percentage of it) is a more sensitive measure for excessive pulsatility transmission to the brain compared to conventional measures such as pressure and flow pulsatility indices alone.

Transmission of arterial pulsatility to the brain has long been known to promote vascular events such as ischemic and hemorrhagic stroke. More recently, hypertension has been shown to promote dementia, accounting for up to 30% of cases^43^. The hemodynamic mechanisms underlying these associations have not been well characterized. Aortic stiffening is the primary cause of systolic hypertension with aging^44^. In our prior work we have shown that the aortic arch stiffening that occurs with aging^45^ is a much more powerful predictor of insult to the microvasculature in the brain than blood pressure or the presence of hypertension treatment^14^. Our results affirm that aortic stiffening does indeed increase transmission of harmful pulsatility to the brain. More importantly we also saw that this increase was several folds more severe when other parameters such as HR also become suboptimal. Results from simulations have been compared to published clinical data in order to verify the clinical relevancy of the computational model (Fig. 3). As it has been demonstrated, the calculated carotid flow augmentation index and pressure augmentation index from simulation data are well within the range of clinical data, and follow similar trends. This confirms the physiological accuracy of our study for purposes of investigating pulsatility transmission to the cerebral vasculature. This may help us identify with much greater accuracy those persons at risk of cerebrovascular events and accelerated brain aging due to harmful effects of excessive arterial pulsatility.

We studied the effect of aortic arch stiffening on the pulsatile energy transmission to the brain across a physiological range of HRs while keeping CO(=5.2 L/min) and other vascular parameters constant. Our results show that there is an optimum HR at which the transmitted pulsatile energy to the brain is minimized (Fig. 4(a)). The pulsatile energy decreases with increasing HR until it reaches this minimum value. Beyond the value, waves transmitted to the brain start acting destructively and, as a result, the pulsatile power starts elevating as HR increases. The existence of an optimum wave reflection has been shown in different contexts related to ventricular workload in animals^21,46^, *in-vitro* experimental data^21^, and computational data^22^. Results are consistent with previous studies that have suggested the idea that aortic wave optimization is one of the design characteristics found in the mammalian cardiovascular system^21^. However, the connection of the wave optimization in a heart-brain coupling framework has been thus far unknown. In this work, we have demonstrated (Fig. 5) the presence of an optimum wave condition —found near the normal human heart beat—for the transmitted energy to the brain across different aortic arch rigidities and HRs.

Unfortunately, as people age and become frail, the resting HR increases^47^ even when it is impaired from appropriately increasing in response to physical exertion^48^. Effects of harmful pulsatile energy transmission to the brain that result from aortic stiffening with aging is likely compounded by a harmful interaction with increased HR among frail elderly at rest and by a decreased HR during exertion. This finding has not been reported before from prior *in-vivo* experiments that involve evaluating arterial stiffness and blood flow for a person at rest. *In vivo*-work fails to capture the impact on cerebral hemodynamics of the changes in HR that occur throughout the day due to physical exertion.

Pressure and flow pulsatility indices (PPI and FPI) are the conventional dimensionless parameters for monitoring hemodynamic pulsatility transmission to the brain. Figure 6 displays results based on the PPI and FPI. As expected, these indices capture the effect of the aortic arch stiffness on pulsatility, and they additionally support the presence of an optimum wave condition when passing from a low HR to a high HR. In other words, increasing or decreasing the HR (the green and blue curves in Fig. 6) will render the transmitted wave suboptimal. Since aortic aging affects the transmitted flow and pressure waves to the brain simultaneously, there is a need to employ a parameter which considers the combined effects of pressure and flow pulsatilities. Hence, we have utilized an energy-based index defined as the ratio of the pulsatile power over the total power transmitted to the brain per cardiac cycle. We have compared the performance of this index with PPI and FPI. The results show that the increase in PPP is much more significant than the other two indices. This suggests that the PPP is more sensitive to changes in wave dynamics and can provide better insight into the effect of aortic arch stiffness and its subsequent behavior on excessive pulsatile power transmission to the brain. Additionally, on the effect of the HR, it has been found that flow pulsatility is more consistent with PPP. Both the FPI as well as the PPP have their highest values at higher HRs (blue curves in Fig. 6), while the PPI has its highest value at lower HRs (green curve in Fig. 6). This work shows that pressure and flow pulsatility may not be considered as interchangeable measures of cerebral pulsatility. A combined consideration of pressure and flow are needed to properly understand the power transmitted to the brain, and future clinical studies should include both assessments.

A limitation of this study lies in the 1D vasculature model formulation. The model used here may not necessarily reveal all aspects of flow distribution in the arterial network, especially those of the cerebral arteries and the morphology of carotid arteries. There are numerous mechanisms that may impact transmission of pulsatility to the brain which are still under investigation. Schubert *et al*.^49^ have recently demonstrated that the contorted shape of the distal internal carotid artery may attenuate the flow pulsatility and hence may provide a protective effect on downstream cerebral vasculature. In another study, Seong *et al*.^50^ investigated the dilation of carotid sinus to reduce the blood flow pulsatility to protect the brain. Further three-dimensional modeling and phase contrast MR at the skull based should be done to investigate these phenomena. In addition, a detailed exploration of how pulsatility is conveyed in the brain and the effect of the circle of Willis on the transmitted pulsatility to the brain are beyond the scope of the current work. In this work, we have focused on the interaction between the aorta and the cerebral circulation; the model has demonstrated capability of capturing the main features of flow and pressure wave pulsatility in larger arteries (Fig. 2). Therefore, it is a good starting point for investigating the effects of arch stiffness on transmitted pulsatility to the brain. Future works involves studying the widespread variability in the circle of Willis and its potential effects on the transmission of arterial pulsatility into different cerebrovascular beds.

Additionally, employing a linear model to describe the dynamics of vessel walls may introduce errors relating to the wall stress relaxation. However, it has been shown previously that, under normal physiological conditions, this error is not very significant^33^. A further limitation of the current model is the approximation of the dynamics of the heart as a flow source imposed as a flow wave at the inlet. Although in general the heart is neither a flow nor pressure source, the behavior of the normal heart is closer to a flow source, and this interpretation has been employed in literature to validate the 1D model and has been shown to be a reasonable approximation^21,22^.

## Conclusion

We have demonstrated that at different aortic arch rigidities, there is an optimum wave condition that minimizes the pulsatile energy transmitted to the brain. This optimum condition occurs near the normal HR and remains constant across a wide range of aortic arch stiffnesses. Based on an energy-based analysis of the waves at the carotid artery, pulsatile power percentage was used as an index to consider the combined effects of pressure and flow changes as the aortic arch become stiffer. This non-dimensional parameter was compared across different wave conditions with pulsatility indices that are based on pressure and flow. Results demonstrate that pulsatile power percentage can capture the transmitted pulsatility to the brain more clearly than the other pulsatility indices due to a higher sensitivity to different wave conditions. Previous work has discussed pathological waves—defined as abnormalities in aortic and coronary wave dynamics—as a potential trigger towards cardiac death in the presence of the cardiovascular disease^51^. The pathology in the wave dynamics and the detection of wave condition signatures for different aortic arch rigidities may provide further insight into the underlying wave dynamics of the arterial system, particularly for the brain-heart coupling portion of the vasculature. Understanding the physics can potentially be a first step towards contributing in the development of new therapeutic strategies for neurodegenerative diseases like Alzheimer’s dementia.

## Supplementary information

Supplemental information.

Supplemental information.
