# Supplementary material for: Dynamic Effects of Aortic Arch Stiffening on Pulsatile Energy Transmission to Cerebral Vasculature as A Determinant of Brain-Heart Coupling
Source: Sci Rep. 2020 May 29;10:8784. doi: 10.1038/s41598-020-65616-7 (PMC7260194; doi:10.1038/s41598-020-65616-7)
Supplement: Supplementary file 2 — Supplemental information. [file 41598_2020_65616_MOESM2_ESM.docx]

**Dynamic Effects of Aortic Arch Stiffening on Pulsatile Energy Transmission to Cerebral Vasculature as A Determinant of Brain-Heart Coupling**

**Arian Aghilinejad ^1^, Faisal Amlani ^1^, Kevin S. King ^2^, and Niema M. Pahlevan ^1, 2, 3, *^**

^1^ Department of Aerospace and Mechanical Engineering, University of Southern California, Los Angeles, CA, USA

^2^ Huntington Medical Research Institutes, Advanced Imaging Center, Pasadena, CA, USA

^3^ Department of Medicine, Division of Cardiovascular Medicine, University of Southern California, Los Angeles, CA, USA

^*^ Corresponding Author. pahlevan@usc.edu

Contact information of the authors:

Arian Aghilinejad: [aghiline@usc.edu](mailto:aghiline@usc.edu)

Faisal Amlani: [faisal.amlani@usc.edu](mailto:faisal.amlani@usc.edu)

Kevin S. King: [kevin.king@hmri.org](mailto:kevin.king@hmri.org)

Niema M. Pahlevan: [pahlevan@usc.edu](mailto:pahlevan@usc.edu)
